# Supplementary material for: Prevalence of a BRCA2 Pathogenic Variant in Hereditary-Breast-and-Ovarian-Cancer-Syndrome Families with Increased Risk of Pancreatic Cancer in a Restricted Italian Area
Source: Cancers (Basel). 2023 Apr 3;15(7):2132. doi: 10.3390/cancers15072132 (PMC10093547; doi:10.3390/cancers15072132)
Supplement: Supplementary file 1 [file cancers-15-02132-s001.zip › cancers-2297982-supplementary.pdf]

Table S1. Clinical history of family members with disease-associated *BRCA2* pathogenic variant. BC, breast cancer; mBC, male breast cancer; BC IL, BC ipsilateral; BC CL, BC controlateral; OC, ovarian cancer; PDAC, Pancreatic Ductal Adenocarcinoma; PrC, prostate cancer; IPMN, Intraductal Papillary Mucinous Neoplasm; WT wild type; BRCA2 +, PV carrier condition; * no test available; A219 family has additional cases not included in the table.

		Sample ID								
		**01**	**02**	**03**	**04**	**05**	**06**	**07**	**08**	**09**
Family	**A141**	Brca2 +<br>BC<br>PDAC	Brca2 +<br>BC<br>BC CL<br>IPMN	Brca2 +	Brca2 +<br>PDAC	Brca2 +<br>IPMN	Wt	Brca2 +		
	**A213**	Brca2 +<br>BC<br>BC CL	Brca2+<br>BC<br>OC	Brca2 +	Brca2 +<br>BC	Brca2 +<br>BC<br>BC IL	Brca2+<br>BC<br>BC CL	WT	Brca2+	Wt
	**A219**	Brca2 +<br>BC	Brca2 +<br>PDAC	Brca2 +<br>PDAC	Brca2 +<br>BC	Brca2 +	Brca2+<br>BC	Brca2 +	wt<br>BC	Brca2+<br>BC
	**A325**	Brca2 +<br>BC	Brca2 +<br>PrC<br>mBC<br>mBC CL<br>IPMN	Brca2 +	WT	Brca2 +<br>BC	Brca2+	WT		
	**A597**	Brca2 +	Brca2 +<br>OC	WT	WT	WT	WT			
	**A726**	Brca2 +<br>BC<br>BC CL<br>OC	Brca2 +<br>OC<br>PDAC	Brca2 +	OC<br>*					
	**A815**	Brca2 +<br>BC	WT<br>BC	Brca2 +<br>PDAC	Wt	Wt	Wt	BC*		
	**B179**	Brca2 +<br>BC	Brca2 +	Brca2 +	Wt	Wt	Brca2+	Wt		
	**B302**	Brca2 +<br>BC<br>IPMN	Brca2 +<br>BC<br>BC CL<br>IPMN	OC*	Wt	Wt				
	**B717**	Brca2 +<br>BC	Brca2 +	Wt	Brca2 +<br>PDAC	Brca2 +<br>BC<br>BC IL	WT	Brca2 +		
	**B849**	Brca2 +<br>BC<br>BC IL	Brca2 +<br>PrC	BC*<br>WT	Brca2 +<br>BC<br>PDAC	BC*	Brca2+<br>BC	Brca2 +<br>BC	Brca2+	
	**B1731**	Wt	Brca2 +<br>PDAC	Wt	BC*	OC<br>PDAC*				
	**B2000**	Brca2 +<br>BC	Brca2 +<br>mBC							
